# Combined Minimum-Run Resolution IV and Central Composite Design for Optimized Removal of the Tetracycline Drug Over Metal–Organic Framework-Templated Porous Carbon

**DOI:** 10.3390/molecules24101887

**Published:** 2019-05-16

**Authors:** Thuan Van Tran, Duyen Thi Cam Nguyen, Hanh T. N. Le, Long Giang Bach, Dai-Viet N. Vo, Kwon Taek Lim, Linh Xuan Nong, Trinh Duy Nguyen

**Affiliations:** 1 Center of Excellence for Green Energy and Environmental Nanomaterials (CE@GrEEN), Nguyen Tat Thanh University, 300A Nguyen Tat Thanh, District 4, Ho Chi Minh City 755414, Vietnam; tranuv@gmail.com (T.V.T.); ntcamduyen@gmail.com (D.T.C.N.); vietvo@ump.edu.my (D.-V.N.V.); linhviet1510@gmail.com (L.X.N.); 2 NTT Hi-Tech Institute, Nguyen Tat Thanh University, 300A Nguyen Tat Thanh, District 4, Ho Chi Minh City 755414, Vietnam; blgiang@ntt.edu.vn; 3 Department of Pharmacy, Nguyen Tat Thanh University, 298–300A Nguyen Tat Thanh, Ward 13, District 4, Ho Chi Minh City 700000, Vietnam; 4 Institute of Hygiene and Public Health, 159 Hung Phu, Ward 8, District 8, Ho Chi Minh City 700000, Vietnam; lethingochanh@iph.org.vn; 5 Center of Excellence for Functional Polymers and Nano-Engineering, Nguyen Tat Thanh University, 300A Nguyen Tat Thanh, District 4, Ho Chi Minh City 755414, Vietnam; 6 Faculty of Chemical & Natural Resources Engineering, University Malaysia Pahang, Lebuhraya Tun Razak, 26300 Gambang, Kuantan, Pahang, Malaysia; 7 Department of Display Engineering, Pukyong National University, Nam-Gu, Busan 608-737, Korea; ktlim@pknu.ac.kr

**Keywords:** minimum-run resolution IV, central composite design, response surface methodology, metal-organic framework, mesoporous carbon, adsorption mechanisms

## Abstract

In this study, a minimum-run resolution IV and central composite design have been developed to optimize tetracycline removal efficiency over mesoporous carbon derived from the metal-organic framework MIL-53 (Fe) as a self-sacrificial template. Firstly, minimum-run resolution IV, powered by the Design–Expert program, was used as an efficient and reliable screening study for investigating a set of seven factors, these were: tetracycline concentration (A: 5–15 mg/g), dose of mesoporous carbons (MPC) (B: 0.05–0.15 g/L), initial pH level (C: 2–10), contact time (D: 1–3 h), temperature (E: 20–40 °C), shaking speed (F: 150–250 rpm), and Na^+^ ionic strength (G: 10–90 mM) at both low (−1) and high (+1) levels, for investigation of the data ranges. The 20-trial model was analyzed and assessed by Analysis of Variance (ANOVA) data, and diagnostic plots (e.g., the Pareto chart, and half-normal and normal probability plots). Based on minimum-run resolution IV, three factors, including tetracycline concentration (A), dose of MPC (B), and initial pH (C), were selected to carry out the optimization study using a central composite design. The proposed quadratic model was found to be statistically significant at the 95% confidence level due to a low *P*-value (<0.05), high R^2^ (0.9078), and the AP ratio (11.4), along with an abundance of diagnostic plots (3D response surfaces, Cook’s distance, Box-Cox, DFFITS, Leverage versus run, residuals versus runs, and actual versus predicted). Under response surface methodology-optimized conditions (e.g., tetracycline concentration of 1.9 mg/g, MPC dose of 0.15 g/L, and pH level of 3.9), the highest tetracycline removal efficiency via confirmation tests reached up to 98.0%–99.7%. Also, kinetic intraparticle diffusion and isotherm models were systematically studied to interpret how tetracycline molecules were absorbed on an MPC structure. In particular, the adsorption mechanisms including “electrostatic attraction” and “π–π interaction” were proposed.

## 1. Introduction

**Figure 1:**
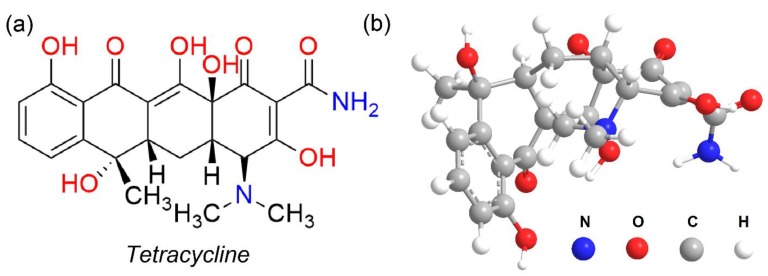
(**a**) Tetracycline (TCC) molecular and (**b**) its simulation structure with the Chem3D program.

Among the most widely consumed antibiotics for infectious diseases, tetracycline (TCC) is a promising candidate, mainly because it exhibits excellent performance as well as cost-effective production [1,2,3] (its simulated molecular and main properties as shown in Figure 1, and Table S1). This antibiotic compound is also used as a food additive for enhancing resistance and supporting the immune system in livestock [4]. Like other antibiotics, TCC can be partly metabolized via organisms, and is mainly excreted through urine [5,6]. Some studies have indicated that a wide range of adverse effects towards both aquatic and soil environments could occur when TCC is released without any pretreated measures [2,3]. For example, Sengelov et al. analyzed the tetracycline, macrolides, and streptomycin contents in Danish farmland, and found a considerable increase in the resistance of bacteria levels in manure and slurry [7]. Moreover, Kay et al. conducted the lysimeter experiments to demonstrate that the fate and occurrence of veterinary antibiotics can derive from surface runoff and leaching ways [8]. Therefore, the remediation techniques for TCC have been increasingly attracting attention over the past years.

The adsorbents with diverse functional groups and high-porosity properties can be used to cope with the TCC antibiotic’s residue [3,9,10]. Zhang et al. proved the TCC removal efficiency (up to 99.8%) of multi-walled carbon nanotubes (MWCNTs) in water, reaching an amazingly high maximum adsorption capacity (269.54 mg/g) at 20 °C [11]. Other carbonaceous sources (e.g., activated carbon) with chemical modifications (e.g., alkaline pretreatment, metal doping, etc.) also brought many promising uptake results for TCC treatment [12,13,14]. Recently, such mesoporous carbons (MPC) can be facilely synthesized via the pyrolysis of metal–organic frameworks (MOFs) as self-sacrificial templates [15,16,17,18]. The MOFs are highly crystalline and porous materials, constructed by iron clusters and organic ligands, giving them excellent tailorability and versatile functionalities [19,20,21,22]. Due to tailoring the electron-rich functionalized adsorbents via these MOFs precursors, applications of MOFs-derived porous carbons (PCs) have been widened, especially in the removal of antibiotics. For instance, Sung et al. utilized a metal-azolate framework-6-derived porous carbon to remove emergent pharmaceutical and personal care products such as ibuprofen, triclosan, oxybenzone, diclofenac sodium, and atenolol [23]. In the same trend, our previous works have also reported the effective utilization of MPC from MIL-53 (Fe) (MIL = Materials Institute Lavoisier) and Fe_3_O(BDC)_3_ (or MIL-88B) for the removal of ciprofloxacin and chloramphenicol with high adsorption capacities [24,25]. As inspired by bifunctional MPC (magnetism for easy separation and efficient adsorbability towards antibiotics), we continued to shift its potential applications to the removal of TCC in water. 

Generally, the optimization procedure is vital to reach the best conditions in adsorption studies, hence, influential factors (e.g., adsorbent dose, concentration, and solution pH) need to be investigated. Unfortunately, there is an abundance of parameters for investigation, resulting in a large number of experimental runs within the laboratory scale [26,27]. Screening studies, therefore, play a crucial role in selecting significantly important parameters but also being eligible to remove insignificant variables [28]. One of the most useful features of screening studies is to limit the actual runs, therefore leading to a noticeable decrease in performance-related costs while the experiments still obtain a high confidence level under standardized tests [29,30,31]. Indeed, these screening studies (e.g., minimum-run resolution IV) are first conducted to shorten the number of factors and the optimization models (e.g., response surface methodology (RSM)) can then be applied for other factors.

Herein, we combined two consecutive procedures, including minimum-run resolution IV as a screening study and central composite design based RSM to optimize the adsorption process of TCC onto MPC. Four out of seven factors (concentration, dose, pH, contact time, temperature, shaking speed, and ionic strength) were eliminated after applying a minimum-run resolution IV as a preliminary probe, and the other factors continued to be optimized using the central composite design approach again.

## 2. Experimental Section

### 2.1. Chemicals, Analytical Instruments, and the Synthesis Procedure for MIL-53 (Fe) and MPC Materials

**Figure 2:**
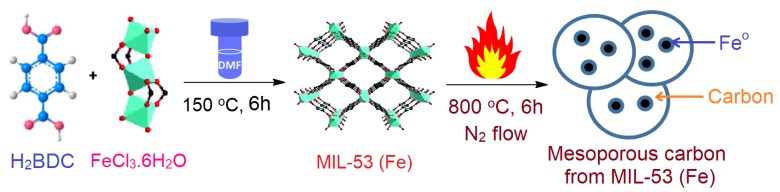
Synthesis route of MIL-53 (Fe) and its pyrolysis product, mesoporous carbon (MPC). The simulated structures of MIL-53 (Fe) and iron (III) clusters were reproduced from a reference [32].

Chemicals, instruments, and the synthesis procedure for MIL-53 (Fe) and MPC materials are described in the supplementary information (SI) materials. In addition, nonlinear adsorption kinetic, isotherm equations, and the mathematical formula are addressed and explained in detail. Figure 2 illustrates the schematic route for the synthesis of MIL-53 (Fe) and MPC.

### 2.2. Experimental Batches

Experimental batches were conducted using a shaking machine. For the screening study, seven parameters, including concentration (5–15 mg/L), MPC dose (0.05–0.15 g/L), solution pH (4–8), contact time (1–3 h), temperature (20–40 °C), shaking speed (150–250 rpm), and ionic strength (10–90 mM), were selected to investigate.

For the adsorption kinetics, MPC (0.1 g/L) was poured into 50 mL of TCC solution (10–40 mg/L) at pH 4, and then placed in the shaking tables (200 rpm) at room temperature (25 ± 2 ^°^C). After the regular time intervals (0, 10, 30, 60, 90, 120, 150, 180, and 240 min), the TCC samples were taken to analyze the kinetic concentrations by UV-Vis spectroscopy.

For adsorption isotherms, a similar procedure was employed. A range of TCC concentrations (10–40 mg/L) was investigated at room temperature (25 ± 2 °C) until reaching the equilibrium at 240 min. The formulas for removal *Y* (%) and adsorption capacity *Q* (mg/g) are listed in the SI material (Equation S1, S2).

### 2.3. Screening Study With Minimum-Run Resolution IV

The screening of the effects can initiate multivariate optimization, giving the purpose of selecting the main factors [33]. Among screening studies, such as fractional factorial design (FFD), Taguchi design (TD), and Plackett–Burman design (PBD), minimum-run resolution IV is of great importance in experimental design to facilitate and accelerate the removal of antibiotics [34]. Herein, we determined a set of seven factors (Table 1), which may affect the percentage of TCC, including concentration (A), MPC dose (B), solution pH (C), contact time (D), temperature (E), shaking speed (F), and ionic strength (G). Three levels (–1, 0, +1) were selected to investigate the effect of factors on the percentage of TCC removal (Y). Based on the guides from a minimum-run resolution IV design, there were 20 trials involving 16 factorial points and 4 central (repeated) points.

To ensure the randomization of each experiment, runs were independently conducted in a separate block. The Design-Expert® Software, Version 10 (DX10), from Stat-Ease, Inc. (Minneapolis, MN, USA) was used as a means of data analysis.

### 2.4. Optimization Study With Central Composite Design

After applying the screening effects, the optimization study can be performed upon three of the most influential variables, e.g., concentration (A), MPC dose (B), and solution pH (C). During this step, the central composite design was used to design the space of the experiments (Table 2). According to the guides from this procedure, the two-order polynomial equation needs to be established to show the relationship between the response (Y) and the three variables (X), as in the following equation (Equation 1) [35]:

(1) $$Y=\beta_{o}+\sum_{i=1}^{k}\beta_{i}X_{i}+\sum_{i=1}^{k}\sum_{j=1}^{k}\beta_{ij}X_{i}X_{j}+\sum_{i=1}^{k}\beta_{ii}X_{i}^{2}$$

(2) $$N=2^{k}+2k+c$$

where *y* is the predicted response, and *x*_i_ and *x*_j_ are the independent variables (*i, j* = 1, 2, 3, 4, …k). The parameter *β*_o_ is the model constant, *β*_i_ is the linear coefficient, *β*_ii_ is the second-order coefficient, and *β*_ij_ is the interaction coefficient. The total number of experiments is defined by Equation 2, and in this circumstance, the figure is 20 for k = 3 (three variables investigated). The Design-Expert® Software, Version 10 (DX10), from Stat-Ease, Inc. (Minneapolis, MN, USA) was again used as a means of data analysis.

## 3. Results and Discussion

### 3.1. Characterization of MIL-53 (Fe) and MPC

The crystalline patterns of MIL-53 (Fe) and MPC materials are shown in Figure S1 (see the Supplementary Materials document file). As can be seen in Figure S1a, three typical peaks presented at around 9.6^°^(101), 18.6^°^ (002), and 28.1° (302), presenting for the MIL-53 (Fe) was mostly commensurate with our previous study and several recent works [36,37,38]. This result indicated the successful synthesis of MIL-53 (Fe) via the solvothermal method. Figure S1b displayed an emergent peak at around 45^°^ (110) and a narrow peak at around 35.5^°^ (100), confirming the presence of zero-valent Fe (JCPDS No. 65–4899) in the inherent structure of MPC [39]. More specifically, the broadband between 20^°^ and 30^°^ may be attributable to graphitic carbon formed by the pyrolysis of MIL-53 (Fe) [40]. The Raman spectra in Figure S2 show more identification of MIL-53 (Fe) and MPC. Functional groups are shown in Figure S2a: C-H (615, 875 cm^−1^) and C-C bonds (1160 cm^−1^) of benzene rings, O-C-O bonds (1450, 1504 cm^−1^) of acid groups support the structural consolidation of MIL-53 (Fe), which is generated by Fe clusters and C_6_H_4_(COOH)_2_ ligands [24]. Meanwhile, the exposure of two bands (D and G) in Figure S2b also indicated the existence of graphitic carbon in MPC.

**Figure 3:**
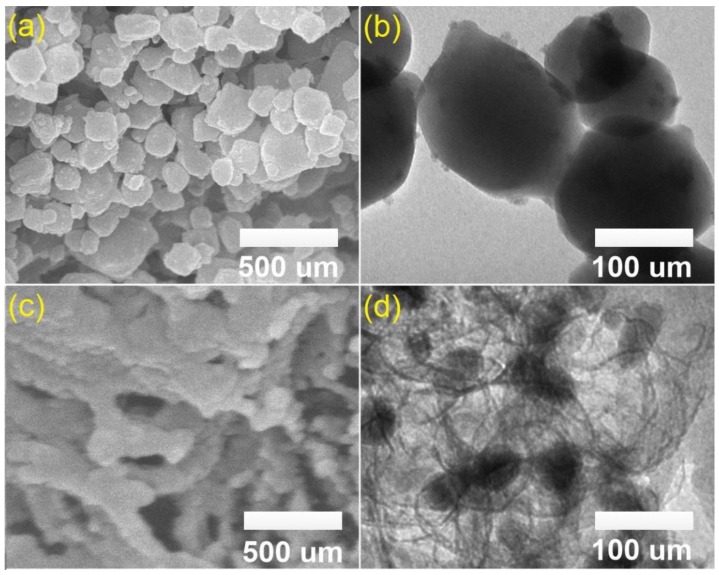
SEM (**a**, **c**) and TEM (**b**, **d**) images of MIL-53 (Fe) (**a**, **b**) and MPC (**c**, **d**).

To gain more understanding about the intrinsic structure of MIL-53 (Fe) and MPC, scanning electron microscope (SEM) and transmission electron microscopy (TEM) images can be solid evidence. As shown in Figure 3a,b, MIL-53 (Fe) crystals were observed like microsphere (100–150 nm in diameter) with a highly smooth surface. By contrast, MPC morphology exposed a relatively defective and amorphous nature, along with the presence of dark spots collapsed in opaque regions. This phenomenon may be because of Fe nanoparticles under magnetic aggregation embedded in carbon [24]. Moreover, the N_2_ adsorption/desorption isotherm plots in Figure S3 give more information about the porosity of MIL-53 (Fe) and MPC. It is evident that MIL-53 (Fe) had a non-porous structure with a Type IV (IUPAC)-like pattern, as shown in Figure S3a, while a hysteresis loop at a higher-relative pressure (P/P^°^ > 0.5) assumed a Type II (IUPAC) pattern with a mesoporous structure. Generally, a huge distinction in structure was observed between MIL-53 (Fe) and MPC upon the effect of pyrolysis. MPC material with a more porous structure (surface area by Brunauer–Emmett–Teller (BET) was approximately 225 m^2^/g) may provide a better advantage in the sorption of TCC. Therefore, we used MPC as an adsorbent for the TCC adsorption investigation.

### 3.2. Screening Study

In the screening study, the possible parameters consisted of the initial TCC concentration (A), the dose of MPC (B), initial pH levels (C), contact time (D), temperature (E), shaking speed (F), and Na^+^ ionic strength (G), as shown in Table 1. In addition, the parameters were represented at three levels, including low (−1), central (0), and high (+1) points (Table 1). Thus, there were 16 preliminary experiments (entries 1–16, Table 3) with a duplicate for each (n = 2). To assess the potential curvature, four central points were repeated (entries 17–20, Table 3). The response (Y) was denoted for the removal percentage of TCC using the MPC as an adsorbent. Each experiment of TCC adsorption was separately conducted based on the guides of minimum-run resolution IV.

By establishing the two-level factorial interaction regression (2FI) with the DX10 program, the ANOVA table allows the observed and predicted data to be analyzed [41,42]. In detail, according to Table 4, estimated effects and their coefficients for two models were listed to find out the significance of parameters at three states, including a significant positive effect at *P* < 0.05, a significant negative effect at *P* < 0.05, and not significant at *P* < 0.05. Apparently, there were four parameters, concentration (A), dose (B), pH (C), and contact time (D), along with a response variable (Y), which were statistically significant, while the other three parameters, temperature (E), shaking speed (F), and Na^+^ ionic strength (G), were not statistically significant (Table 4). Therefore, the latter factors can be eliminated in the next investigation. Moreover, among the four significant parameters, concentration (A) was the only significantly negative effect at *P* < 0.05, while the others, along with the response variable (Y), were the significantly positive effects, indicating that decreasing the concentration, and rising the dose (B), pH (C), and contact time (D) tended to improve the removal percentage of TCC. Moreover, Figure S4 was also constructed to support the high compatibility of the 2FI model [41]. While the residuals against runs plot suggested a random distribution without any patterns, as shown in Figure S4a, the actual and predicted results were mostly distributed in a straight line, as shown in Figure S4b.

**Figure 4:**
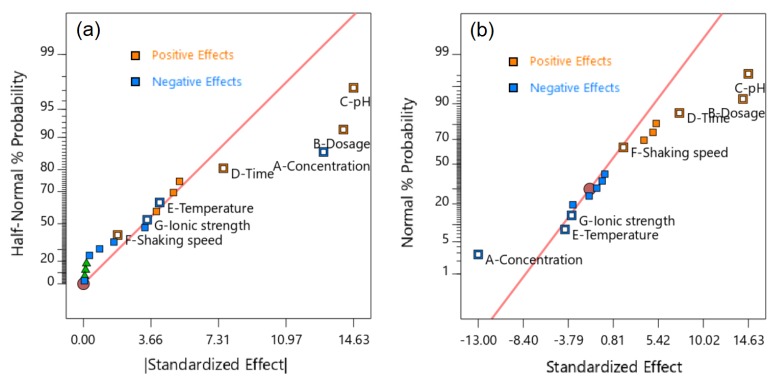
Half-normal (**a**) and normal (**b**) probability plots for seven factors (A– Concentration, B– Dose, C– Solution pH, D– Contact time, E– Temperature, F– Shaking speed, and G– Ionic strength).

**Figure 5:**
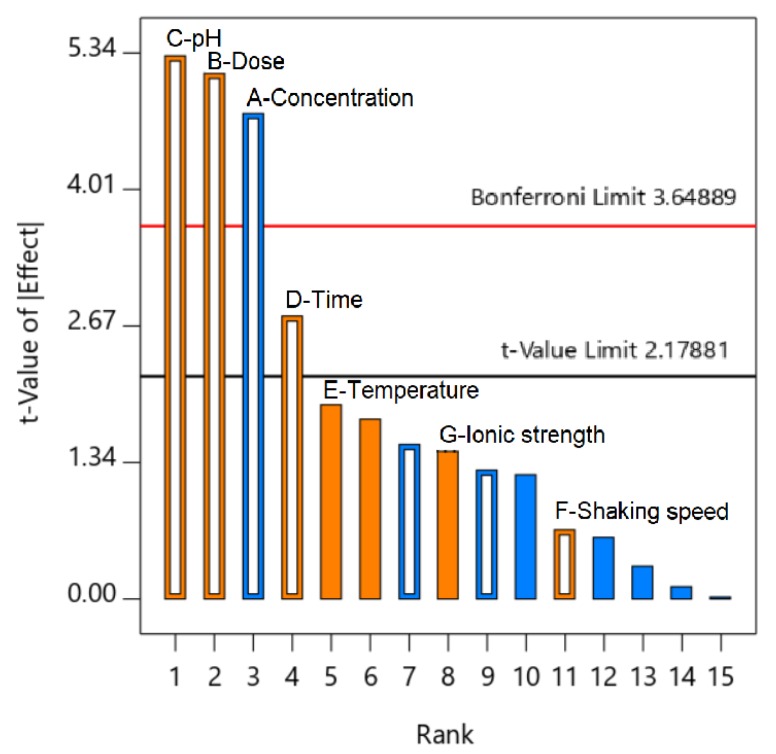
Pareto chart of the standardized effects (A– Concentration, B– Dose, C– Solution pH, D– Contact time, E– Temperature, F– Shaking speed, and G– Ionic strength).

The diagnostic plots also supported the evidence of the significance of the selected factors. For half-normal and normal probability plots for the seven factors (Figure 4), statistically insignificant factors are those that have linear lines near effects, and statistically significant factors are those whose effects are considerably escaped from linear lines [43,44], which were determined to be concentration (A), dose (B), pH (C), and contact time (D). Meanwhile, a Pareto chart of the standardized effects (Figure 5), and a residuals versus runs for the models (Figure 3) shows that the t-values of effects (concentration, dosage, and pH) are higher than the “Bonferroni limit” and higher than those of other effects (contact time, temperature, shaking speed, and Na^+^ ionic strength), suggesting that the most influential factors were concentration, dosage, and pH for the proposed 2FI model [45,46]. Based on the above analysis, the three most influential factors, TCC concentration (A), dose (B), and pH (C), were selected for the further optimization studies using a central composite design, while the other insignificant parameters were neglected.

### 3.3. Optimization Study

After eliminating four factors using the screening study, a central composite design analysis was used to make the experimental space with three factors, TCC concentration (A: 1.6–18.4 mg/L), MPC dose (B: 0.016–0.184 g/L), and pH (C: 2.6–9.4), as shown in Table 2. By establishing the quadratic regression model, the second-order polynomial equation can be generated to evaluate the interactive effect of factors, and then optimize the conditions for the removal of TCC. Under optimized conditions, a confirmation test was performed to check the suitability of the proposed model. All steps for optimization study were taken as follows.

More specifically, Table S2 lists the twenty-trial actual and predicted values for the TCC removal. The highest percentage of TCC removal was 93.0% (entry 3), while the lowest was only 53.0% (entry 14) and the average six trials were 79.8% (entries 15–20). The empirical relationships between the response (Y) and the significant factors (A, B, and C) were achieved from using a central composite design via the Design-Expert® Software [47]:

Y (%) = 79.83 − 3.35A + 7.17B − 8.82C + 1.38AB + 0.125 AC − 2.38BC + 0.41A^2^ − 3.31B^2^ − 3.66C^2^ (3)

Based on these inputs, significant coefficients such as P values, R^2^ coefficients, and AP ratios can be determined from the ANOVA data (Table 5) [48,49]. Generally, a quadratic model is considered statistically significant at a confidence level of 0.95, if it meets the following conditions as closely as possible: P values for models and factors are lower than 0.05, the coefficient of determination (R^2^) is closer to 1.0, and the AP ratio is higher than 4.0 [50]. By comparing the values obtained from Table 5 with the above standards, it is evident that the proposed model was statistically significant with the confidence level at 95%.

Moreover, the residual analysis was also used to confirm the assumption of significance for the proposed model with the three factors (Figures S5–S8). More specifically, the normal plot of residuals in Figure S5a tends to be an “S-shape” rather than linear or a normal line. Nevertheless, Grace et al. indicated that this plot might supply a better analysis with a transformation of the response, meaning that the residuals have been divided by the estimated standard deviation of each particular residual [51]. The predicted versus residuals plot in Figure S5b shows a random scatter, hence, the variance is a constant against the residuals’ variables. This analysis was highly suitable for the residual plot in Figure S6. Accordingly, Figure S6a diagnosed a random distribution without any patterns or trends, while the actual and predicted data in Figure S6b had high-compatibility because these points were randomly scattered along the 45-degree line. Also, Cook’s distance in Figure S7a was used to record any changes made by the quadratic model in the case of deleting or omitting any data point [52]. Although the Cook’s distance of two points was found to be larger than 1.0, which may lead to a lack of their accuracy, the others (18 data points) were lower than 1.0, meaning that omitting one of them is highly unlikely to vary the estimate of the regression coefficients [53]. The Box-Cox plot for power transforms in Figure S7b implies that the current transformation (blue line) was not in the range of the best lambda value (red line), indicating that the current transformation is not required [54]. Figure S8a shows leverage versus run values at two sides with upper and lower the average leverage (0.5), but close to zero, revealing that the clustering of points may be acceptable [51]. Meanwhile, DFFITS is a factor that allows for the determining of significant runs in Figure S8b. Generally, most of the points were inside two limits (±2.12132), indicating an insignificant difference in fitness [51]. Consequently, with the above analysis, the model for TCC removal over MPC reached a high-compatibility with the actual data at the 95% confidence level and could be used to assess the interaction among factors.

**Figure 6:**
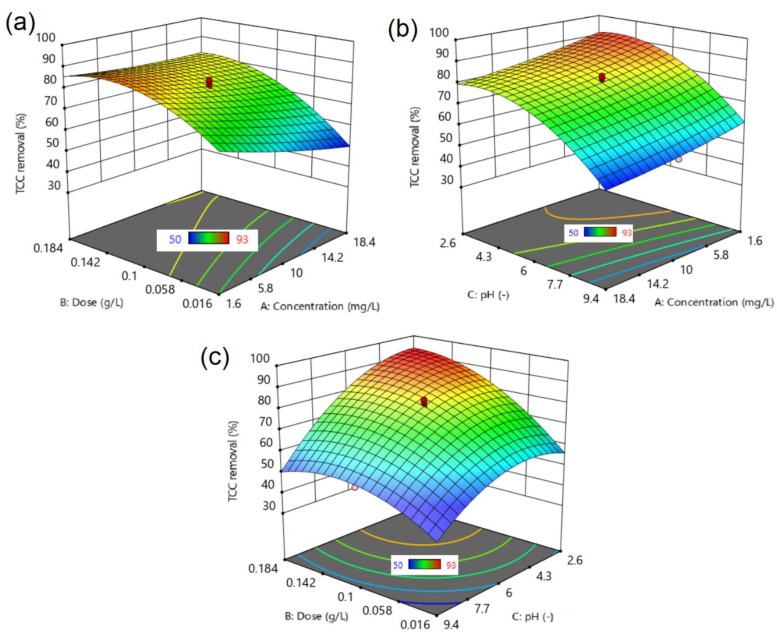
Surface response plots: (**a**) effect of concentration (A) and dose (B); (**b**) effect of concentration (A) and solution pH (C); and (**c**) effect of dose (B) and solution pH (C) on the removal of TCC over MPC.

Three-dimensional (3D) response surface plots, which described the effect of two factors (another was kept at zero-level) on the removal percentage of TCC, are presented in Figure 6. All of the figures show the significant interactions of the factors and led to changes in TCC removal efficiency.

In detail, Figure 6a indicates the effect of concentration (A) and dose (B) on the removal of TCC over MPC. An increase in dose and a decrease in concentration is likely to enhance the overall removal yield. Meanwhile, pH is the most influential factor in Figure 6b, with the best conditions for the removal of TCC being at a low level of pH. Indeed, it is observed that the optimum pH level was in the range from 2.6–6.0, while the effect of concentration is negligible. Liang et al. also reported the same effect of pH on the TCC removal efficiency by organic acid-coated magnetic nanoparticles [55]. In the opposite trend, Figure 6c shows that both dose and pH factors had a strong interaction together, and their resonance vales could bring the best removal efficiency. At a high dose (e.g., 0.184 g/L), and a low pH level (e.g., 4.0), nearly 100% of the TCC could be removed by MPC. For further analysis, the optimized conditions were recommended via a central composite design powered by the DX10, which listed the best options along with the respective desirability values to obtain the highest percentage of TCC removal. Indeed, Table S3 lists three independent runs according to the proposed conditions. It is evident that all of the confirmation tests gave the actual results as equivalent to the proposed ones, with very low errors, again indicating the excellent compatibility between the proposed and the actual model [56]. Nearly 100% of the TCC was eliminated under the optimized conditions (entries 1–3, Table S3). These results proved the promising application of MPC for the separation of TCC from wastewater.

### 3.4. Proposed Adsorption Mechanism

**Figure 7:**
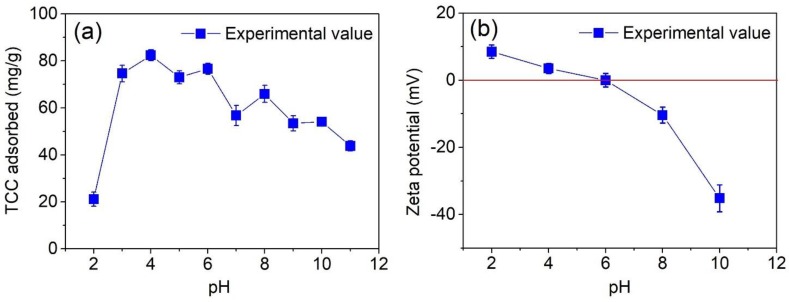
Effect of pH on the TCC adsorption capacity (**a**) and zeta potential of MPC (**b**).

According to the screening (e.g., the Pareto chart) and optimization (e.g., response surfaces) studies, pH was the most influential factor affecting the adsorption of TCC, and thus, the investigation of pH solution was required to gain more insight into how the TCC molecules are absorbed on the MPC surface. Firstly, we fluctuated the pH values (adjusted by using HCl and NaOH solutions) in the wide range from 2 to 11 to observe the change in TCC adsorbed by MPC material, as shown in Figure 7.

The results shown in Figure 7a reveal that the adsorption capacity of the TCC antibiotic reached a peak of 82.4 mg/g at pH 4, while the lowest value was found to be 21.1 mg/g at pH 2. Generally, the adsorption of TCC tended to deplete, thereby increasing the pH solution from 3 to 10. It is also observed that this uptake seemed to be conducive in weak acidic media (3 ≤ pH ≤ 6) rather than in neutral or weak basic solutions (pH ≥ 7). These results were highly in-line with the optimized conditions obtained by the RSM model and confirmation tests (Table S3), as well as with recent publications [57,58,59].

To propose a plausible mechanism of how TCC molecules are adsorbed on the surface of MPC, we determined the effect of pH on the zeta potential of the adsorbent. According to the Figure 7b, increasing the pH level of the solution would lead to a decrease in the magnitude of the zeta potential. Especially at around pH 6, the zeta potential of MPC reached a zero-value or an isoelectric point (IEP_MPC_ = 6), which means that the surface of the MPC tended to be negatively charged under the condition at pH > IEP_MPC_ = 6, and it charged positively at pH < 6. Adsorption of TCC on the surface of MPC may be contributed to by several factors, including electrostatic interaction, hydrogen bonds, and π–π interaction.

It was reported that TCC molecules have three values of acid dissociation constant with pKa_1_ = 3.3, pKa_2_ = 7.7, and pKa_3_ = 9.7 (Table S1). Notably, owing to the protonation or deprotonation process in water, it can exist in various states of ionic species at different pH points [60,61]. Kang et al. demonstrated that TCC molecules (H_2_TCC) at pH values from 1 to 14 can present a wide range of ionic or neutral states as cations (H_3_TCC^+^), zwitterions (H_2_TCC), and anions (HTCC^-^ and TCC^2−^) [62].

At pH < pKa_1_ = 3.3, the surface of MPC becomes charged positively due to pH < IEP_MPC_, thus TCC molecules are immediately protonated to form the cations (TCCH_3_^+^), leading to an opposite charge between adsorbent and adsorbate [63]. As a result, both components possibly appear in an “electrostatic repulsion” force [64], preventing the contact between TCCH_3_^+^ ions and MPC material, and finally, causing a considerable decrease in the adsorption capacity. Supporting this trend, Figure 7a shows a low TCC adsorption capacity (21.1 mg/g) at pH 2, but when the pH values rose, the adsorbability of the MPC towards the TCC molecules increased noticeably. For example, the highest adsorption capacity (82.4 mg/g) in this case was found at pH 4. This phenomenon may be attributable to the fact that TCC molecules are partly deprotonated for the first ionization (TCCH_2_^±^) at pKa_1_ < pH < IEP_MPC_, leading to the generation of an electrostatic attraction between the deprotonated TCC and the MPC surface and boosting the adsorption capacity, as reported by Liu et al. [65]. Marzbali et al. also explained that when a negatively charged adsorbent surface increases gradually, the “electrostatic interaction” becomes stronger, resulting in intermolecular hydrogen bond forces, and increasing adsorption capacity [63].

When the pH gradually reached IEP_MPC_ (pH 6), the surface of the MPC became more neutral, whereas there was no tetracycline ionization (zwitterions) at pH levels between 5 and 6, as reported by Kang et al [62]. As a result, the effect of the “electrostatic interaction” tended to be negligible, causing a noticeable depletion in the TCC adsorption capacity. However, it was observed that the adsorption capacity still remained very high, for example, approximately 80 mg/g of TCC was adsorbed on the MPC at pH 6. Therefore, other main factors can play a crucial role in maintaining the adsorbability of MPC at the pH levels 5–6. Accordingly, both the TCC molecules and the MPC surface own π electrons on the benzene rings, generating the same type of intermolecular electron “donor–acceptor” interaction (or called the π–π interaction) as was reported by Esra et al. and Marzbali et al. [63,66]. The nature of this force relies on a non-hydrophobic interaction between electron-rich benzene rings in the TCC structure and the polarized aromatic rings in the carbonaceous adsorbent [63], which are driving forces for the TCC antibiotic adsorption on MPC materials. Ghadim et al. also interpreted the same enhancement in adsorbability towards nonionic TCC at such a pH region, via the π-π interaction [67].

Under basic solutions, there was a downward trend in the adsorption capacity, probably due to “electrostatic repulsion”, which was formed between the negatively charged MPC surface (pH > IEP_MPC_) and the TCC anions (HTCC^-^ and TCC^2−^) [63]. This hypothesis was consolidated based on the results from Figure 7b, which shows lower-capacity values than those at the acidic region.

### 3.5. Adsorption Kinetics

According to the screening study, the Pareto chart in Figure 5 indicated that contact time is the fourth most influential factor among the seven surveyed factors, so the effect of contact time on the adsorption kinetic needs to be investigated. In addition, based on investigating the effect of pH in Figure 7a, the solutions for the kinetic experiments were adjusted at an optimized pH 4. To begin, finding out the relationship between contact time and kinetic adsorption capacity can be achieved by placing the experiments at four concentrations (10, 20, 30, and 40 mg/L) and determining the TCC concentration by UV-Vis spectroscopy at regular periods.

**Figure 8:**
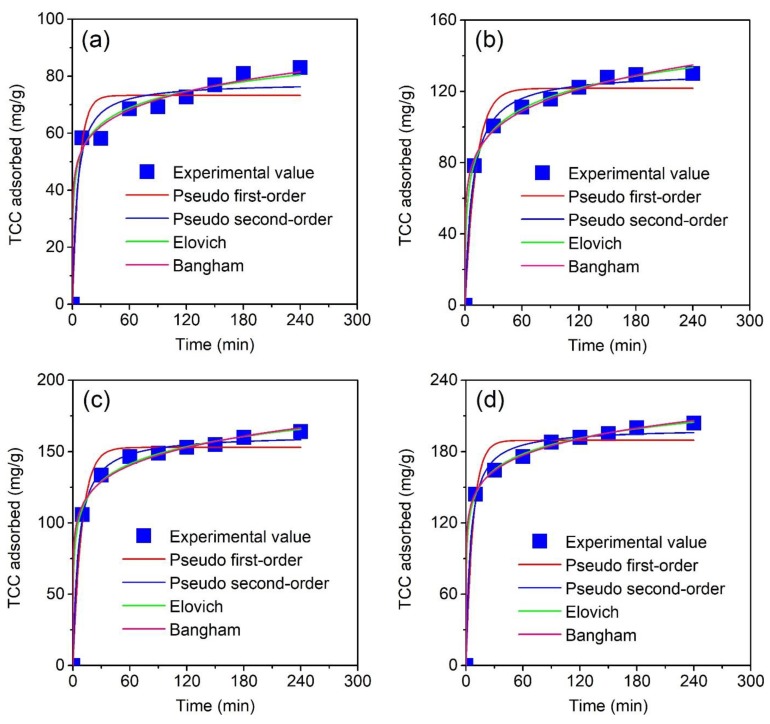
Effect of contact time (0–240 min) at various concentrations (10–40 mg/L) on the TCC adsorption capacity of MPC material. Four nonlinear kinetic models (Pseudo first-order, Pseudo second-order, Elovich, and Bangham) were used to fit the experimental data.

In the present study, we selected the intervals (0, 10, 30, 60, 90, 120, 150, 180, and 240 min) under the constant temperature. According to Figure 8a–d, boosting the contact time led to an increase in adsorption capacity for all plots. Moreover, larger adsorption capacities can be obtained under higher concentrations. Typically, the first 60-minute stage witnessed a sharp rise in the adsorption capacity. Next, the plots tended to reach a steadily and slowly increasing process, and finally, obtained an equilibrium-nature within 240 minutes. Therefore, further experiments could be conducted during this period. To describe the adsorption laws, we applied four non-linear kinetic models consisting of pseudo first-order, pseudo second-order, Elovich and Bangham equations.

Note that the mathematical description for these four models and error functions (R^2^, MRE, and SSE) are available in the Supplementary Materials document file. Theoretically, any model which obtained the standards: (1) most R^2^ values close to 1 (adjusted R^2^ > 0.9), and/or (2) MRE (%) values decrease to zero (MRE < 10%), and/or (3) obtain the lowest SSE values, is mathematically well-fitted, can reflect the relationship between the actual and the proposed data.

According to Table 6, all nonlinear models obtained the excellence-of-fitness based on the adjusted determination of coefficients (adjusted R^2^). Indeed, the adjusted R^2^ values (0.9079–0.9996) reached close to 1.0, along with lower MRE (0.54–8.46) and SSE values. Pseudo first-order models may be unsuitable for describing the actual data because of their lowest adjusted R^2^ (0.9079–0.9770), and greatest MRE (4.23–8.46) and SSE (410.98–1205.53) values. These results were highly agreeable with previous works [55,57,68,69,70]. The others reflected better compatibility based on the error functions. However, the adsorption of TCC over MPC at various concentrations obeyed the Elovich model due to obtaining the highest adjusted R^2^ (0.9839–0.9996)_,_ and the lowest MRE (0.54–3.85%) and SSE (11.71–72.06) among these kinetic models. These results were also commensurate with a recent study on the adsorption of TCC onto NaOH-activated carbon derived from macadamia-nut shells [60]. Therefore, adsorption of TCC adheres to a heterogeneous mechanism, which is neither the desorption or interactions between adsorbed species at low surface coverage [60]. Clearly, according to the Elovich equation, the adsorption rates (α = 536.46–3503.35 mg/g min) were far higher than the desorption rates (β = 0.05–0.12 g/mg), reflecting the absolute dominance of TCC adsorption over MPC.

### 3.6. Intraparticle Diffusion

**Figure 9:**
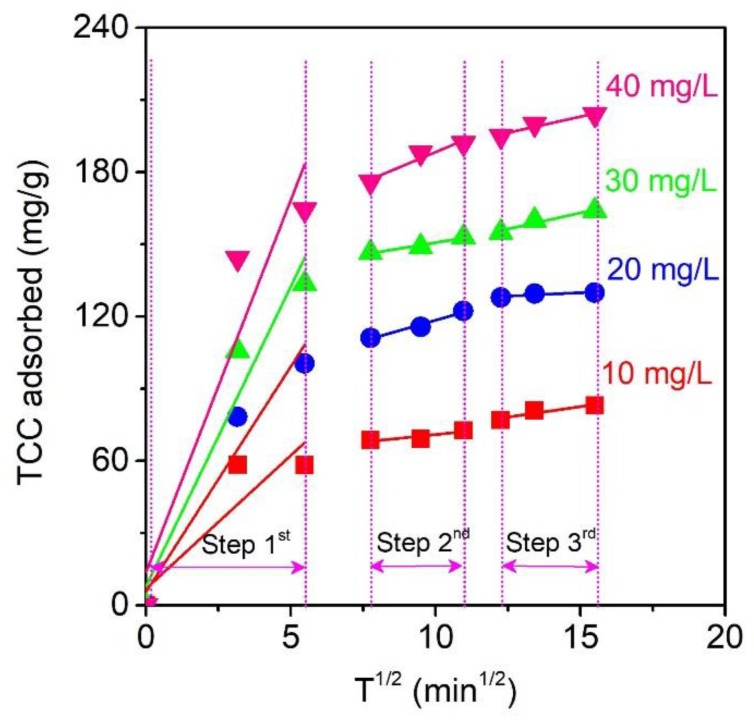
Intraparticle diffusion plots for TCC adsorption over MPC at various concentrations (10–40 mg/L) with three stages: Stage 1 (0–30 min), Stage 2 (60–120 min), and Stage 3 (150–240 min).

To gain insight into the actual rate-controlling step for the TCC adsorption model, the Weber–Morris intraparticle diffusion equation, which assumes that the mechanism for TCC adsorption occurs in the bulk external-mass transfer, or the diffusion of TCC molecules through the micropores of MPC material and chemical reactions (adsorption/desorption) in heterogeneous phrases, could be adopted [60]. Herein, Figure 9 describes the intraparticle diffusion plots for TCC adsorption over MPC at various concentrations (10–40 mg/L)

According to the Weber–Morris plots at four concentrations (10–40 mg/L) in Figure 9, the intraparticle diffusion of TCC over MPC could be divided into three stages. The first stage (0–30 min) is the most rapid adsorption stage, which may be due to the diffusion of the TCC molecules into the external surface or peripheral layers of the MPC materials. The high values of K_id,1_ (11.0891–30.9549 mg/g min^1/2^) in Table 7 indicate the enormous rate of the adsorption process. The second stage describes the gradual adsorption during the next 60-min period (from 60 to 120 min) [69]. The high R^2^ values (0.9058–0.9891) obtained by the second stage of the Weber–Morris equation (Table 7) demonstrated that the intraparticle diffusion at the second stage was the rate-limiting step. The final stage (150–240 min) tended to reach the equilibrium-nature with the slow intraparticle diffusion, mainly because of the shallow concentration of TCC remaining in the solution and a large amount of TCC molecules loaded on the micropores of the MPC. This stage had the lowest adsorption rates (K_id,3_ = 0.5728–2.6756 mg/g min^1/2^) (Table 7) and its model intercepts were found to be as unequal as zero, indicating that the rate-limiting step was dominant not due to intraparticle diffusion solely [71].

### 3.7. Adsorption Isotherms

**Figure 10:**
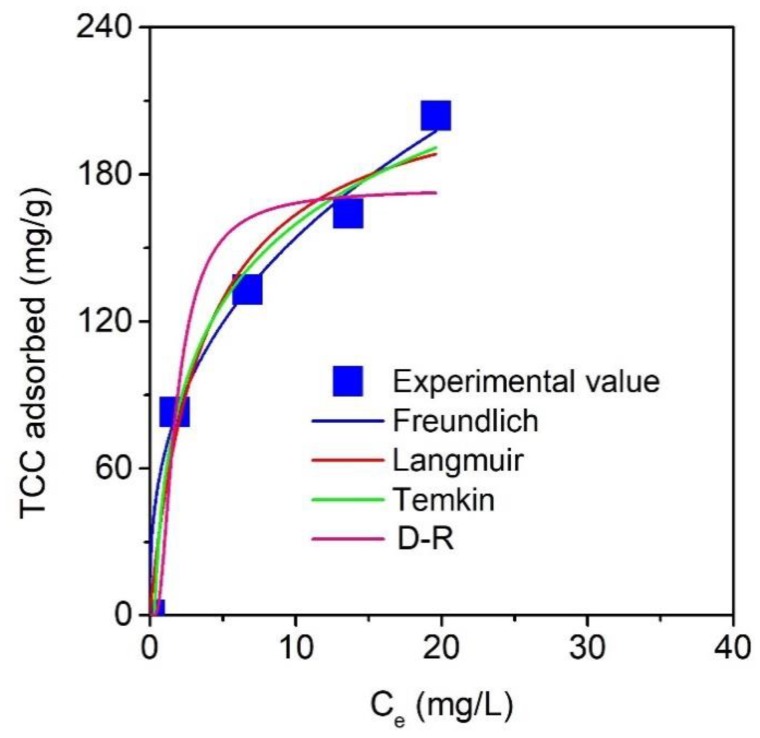
Effect of equilibrium concentration on TCC adsorption uptake. Four non-linear isotherm models (Langmuir, Freundlich, Temkin, and D-R) were used to fit the experimental data.

The nonlinear isotherm models, including Langmuir, Freundlich, Temkin, and Dubinin-Radushkevich (D–R), could be used to better understand the adsorption mechanism and behaviors of TCC over MPC. Note that the mathematical description for their respective parameters and error functions (R^2^, MRE, and SSE) are available in the Supplementary Materials file. Moreover, based on the investigation of the effect of pH in Figure 7a, the solutions for the isotherm experiments were adjusted to an optimized pH 4. Figure 10 describes the effect of concentration (10–40 mg/L) on the equilibrium adsorption capacities (Q_e_, mg/g), which increased the concentration resulting in an improvement in the Q_e_ values.

The isotherm parameters obtained from the equations were summarized in Table 8. Naturally, all models showed excellent fitness, based on the adjusted R^2^ (0.8982–0.9933), and the relatively low MRE (2.95–11.82%) and SSE (124.19–1900.35) values. According to the analysis of these error functions, the order-of-fitness for models is obeyed: Freundlich > Temkin > Langmuir > D–R, suggesting that the mechanism for TCC adsorption over MPC adhered to the Freundlich model, which assumes that multilayer adsorption behavior may be prevalent in this case. Moreover, the exponent value (1/n), which was determined by the Freundlich model, was found to be 0.37, and the R_L_ constant, which was determined by the Langmuir model, was found to be 0.15, suggesting that the adsorption of the TCC molecules over MPC was a favorable process.

### 3.8. Comparative Adsorption Capacity

From the Langmuir equation, the maximum TCC adsorption capacity Q_m_ (mg/g) was calculated as being at 224.0 mg/g. To compare the adsorption capacity obtained by this work with that of other works, we summarized the maximum TCC adsorption capacities of the various materials. The comparative result indicated that the Q_m_ from the present study was considerably higher than those reported from many previous studies (Table 9). Thus, it is recommended that the use of MPC for the adsorption of the TCC antibiotic in water may be a feasible approach.

## 4. Conclusions

The present study successfully synthesized the novel MIL-53(Fe)-derived mesoporous carbon by the pyrolysis of MIL-53 (Fe) and characterized it structurally. The screening study using minimum-run resolution IV was systematically investigated for seven factors, including initial TCC concentration (A), a dose of MPC (B), initial pH (C), contact time (D), temperature (E), shaking speed (F), and Na^+^ ionic strength (G). The results indicated that the most influential factors on the percentage of TCC removal were concentration, MPC dose, and pH solution, based on the ANOVA analysis and diagnostic plots (e.g., the Pareto chart). Next, these factors were used to optimize the percentage of TCC removal through using a central composite design and response surface methodology. It was presented that the proposed quadratic regression model was statistically significant at the 95% confidence level. Under optimized conditions (e.g., TCC concentration of 1.9 mg/g, MPC dose of 0.15 g/L, and pH 4.0), TCC can be eliminated up to 98.0%–99.7%. The results of kinetic and isotherm studies revealed that the adsorption of the TCC drug adhered to the heterogeneous mechanism (Elovich model) and multilayer adsorption behavior (Freundlich model), while intraparticle diffusion of TCC over MPC asserted that the rate-limiting step was not dominant only due to intraparticle diffusion. Under the effect of pH more specifically, the adsorption mechanism, including “electrostatic attraction” and the “π–π interaction”, was proposed in detail. Compared with the other adsorbents, the adsorption of the TCC antibiotic in wastewater by the utilization of MPC can be a feasible approach with the high-maximum adsorption capacity (224 mg/g).

## Figures and Tables

**Table 1:** Summary of influential variables with their levels.

No	Parameter	Unit	Code	Level		
				Low (–1)	Central (0)	High (+1)
1	**Tetracycline (TCC) concentration**	**mg/L**	A	5	10	15
2	Mesoporous carbon (MPC) dose	g/L	B	0.05	0.1	0.15
3	Initial pH	-	C	2	6	10
4	Contact time	h	D	1	2	3
5	Temperature	°C	E	20	30	40
6	Shaking speed	rpm	F	150	200	250
7	Na^+^ ionic strength	mmol/L	G	10	50	90

**Table 2:** List of variables for optimization of TCC removal.

No	Independent Factors	Unit	Code	Levels				
				–α	−1	0	+1	+α
**1**	**Initial concentration**	**mg/L**	A	1.6	5	10	15	18.4
2	MPC dose	g/L	B	0.016	0.05	0.1	0.15	0.184
3	Solution pH	-	C	2.6	4	6	8	9.4

**Table 3:** Twenty-trial minimum-run resolution IV screening design for seven actual values with their observed and predicted values.

Run	Experimental Values							TCC Removal (%)	
	A	B	C	D	E	F	G	Observed	Predicted
1	5	0.05	10	3	20	250	10	90.2	87.8
2	5	0.15	2	3	20	150	90	83	82.0
3	5	0.15	10	3	40	150	10	95.5	95.9
4	5	0.15	2	1	40	250	10	70.3	75.6
5	5	0.05	2	3	40	250	90	63.2	65.6
6	15	0.15	2	3	20	250	10	80.1	74.3
7	5	0.15	10	1	20	250	90	85.7	90.9
8	15	0.15	10	3	40	250	90	85.2	81.3
9	15	0.15	2	1	40	150	90	55.7	57.3
10	15	0.05	10	1	40	250	10	60.2	63.1
11	15	0.05	2	1	20	250	90	52.7	49.2
12	15	0.05	10	3	20	150	90	60.3	69.5
13	15	0.15	10	1	20	150	10	81	79.5
14	5	0.05	10	1	40	150	90	80.6	70.8
15	15	0.05	2	3	40	150	10	53	54.2
16	5	0.05	2	1	20	150	10	63.7	63.8
17	10	0.1	6	2	30	200	50	80.6	72.5
18	10	0.1	6	2	30	200	50	80.3	72.5
19	10	0.1	6	2	30	200	50	82.6	72.5
20	10	0.1	6	2	30	200	50	79.4	72.5

**Table 4:** Estimated effects and their coefficients for TCC models.

Parameters	Code	Effect Estimate	Coefficient Estimate	Standard Error	*F*-value	*P*-value
Response	Y	-	-	1.37	12.79	0.0002 ^a^
Concentration	A	−13.0	−6.5	1.37	22.55	0.0006 ^b^
Dose	B	14.1	7.1	1.37	26.44	0.0003 ^a^
pH	C	14.6	7.3	1.37	28.54	0.0002 ^a^
Contact time	D	7.6	3.8	1.37	7.66	0.0183 ^a^
Temperature	E	−4.1	−2.1	1.37	2.27	0.1600 ^c^
Shaking speed	F	1.9	0.9	1.37	0.46	0.5131 ^c^
Na^+^ ionic strength	G	−3.5	−1.7	1.37	1.59	0.2336 ^c^

^a^ Significantly positive effect at *P* < 0.05. ^b^ Significantly negative effect at *P* < 0.05. ^c^ Not significant at *P* < 0.05.

**Table 5:** ANOVA data for the TCC removal model.

Source	Sum of Squares	Degree of Freedom	Mean Square	*F*-value	Prob. > F	Comments
Model	2311.79	9	256.87	10.94	0.0004 ^s^	SD = 4.85
A	153.72	1	153.72	6.55	0.0284 ^s^	Mean = 75.35
B	701.27	1	701.27	29.87	0.0003 ^s^	CV (%) = 6.43
C	1061.60	1	1061.60	45.22	<0.0001 ^s^	R^2^ = 0.9078
AB	15.12	1	15.12	0.6443	0.4408 ^n^	AP = 11.4
AC	0.1250	1	0.1250	0.0053	0.9433 ^n^	
BC	45.13	1	45.13	1.92	0.1957 ^n^	
A^2^	2.37	1	2.37	0.1012	0.7570 ^n^	
B^2^	157.55	1	157.55	6.71	0.0269 ^s^	
C^2^	193.04	1	193.04	8.22	0.0167 ^s^	
Residuals	234.76	10	23.48	-	-	
Lack-of-fit	212.76	5	42.55	9.67	0.0132 ^s^	
Pure error	2311.79	9	256.87	10.94	0.0004 ^s^	

**Note**: ^s^ significant at *P* < 0.05; ^n^ not significant at *P* > 0.05, SD: standard deviation, CV: coefficient of variation, AP: Adequate precision, R^2^: determination of coefficient.

**Table 6:** Kinetic constants for the TCC adsorption over MPC material at various concentrations.

Kinetic Models	Equation	Parameters	TCC Concentrations			
			10 mg/L	20 mg/L	30 mg/L	40 mg/L
**Pseudo first-order**	$Q_{t}=Q_{1}.\left(1-\exp(-k_{1}t)\right)$	k_1_ (min^-1^/(mg/L)^1/n^)	0.1449	0.0878	0.1079	0.1336
		Q_1_ (mg/g)	73.25	121.71	153.03	189.56
		MRE (%)	8.46	6.96	4.23	5.14
		SSE	410.98	572.74	430.34	1205.53
		(R_adj_)^2^	0.9079	0.9583	0.9770	0.9641
**Pseudo second-order**	$Q_{t}=\frac{t}{\frac{1}{k_{2}Q_{2}^{2}}+\frac{t}{Q_{2}}}$ $H=k_{2}.\;Q_{2}^{2}\;$	k_2_ (g/(mg.min))	0.0027	0.0010	0.0011	0.0011
		Q_2_ (mg/g)	77.80	131.01	162.16	199.57
		H	16.22	16.97	28.40	44.21
		MRE (%)	7.04	3.62	1.86	3.01
		SSE	179.58	147.66	72.01	318.46
		(R_adj_)^2^	0.9496	0.9904	0.9961	0.9889
**Elovich**	$Q_{t}=\frac{1}{\beta }\ln(1+\alpha \beta t)$	α (mg/(g.min))	536.46	196.76	991.85	3503.35
		β (g/mg)	0.12	0.06	0.06	0.05
		MRE (%)	3.85	1.25	1.81	0.54
		SSE	72.06	18.12	73.43	11.71
		(R_adj_)^2^	0.9839	0.9976	0.9961	0.9996
**Bangham**	$Q_{t}=k_{B}.t^{\alpha_{B}}$	k_B_ (mL/(g/L))	40.33	57.96	84.84	113.06
		α_B_	0.13	0.15	0.12	0.11
		MRE (%)	3.03	1.93	2.32	0.67
		SSE	53.86	43.67	120.34	17.69
		(R_adj_)^2^	0.9879	0.9948	0.9936	0.9994

**Table 7:** Parameters of the intraparticle diffusion model with three stages.

Parameters	TCC Concentrations			
	10 mg/L	20 mg/L	30 mg/L	40 mg/L
K_id,1_ (mg/g min^1/2^)	11.0891	18.7447	24.9274	30.9549
C_i,1_ (mg/g)	6.9320	5.6518	8.0132	13.7884
R^2^	0.9058	0.9755	0.9728	0.9487
K_id,2_ (mg/g min^1/2^)	1.2510	3.4347	1.9758	5.0471
C_i,2_ (mg/g)	58.4464	84.1640	130.9694	137.9451
R^2^	0.9161	0.9891	0.9815	0.9717
K_id,3_ (mg/g min^1/2^)	1.7464	0.5728	2.6756	2.6560
C_i,3_ (mg/g)	56.374	121.3084	122.9612	162.9612
R^2^	0.9394	0.9043	0.9750	0.9510

**Table 8:** Isotherm constants for the TCC adsorption over MPC material.

Kinetic Models	Equation	Parameters	Value
Langmuir	$Q_{e}=\frac{Q_{m}K_{L}C_{e}}{1+K_{L}C_{e}}$ $R_{L}=\frac{1}{1+K_{L}C_{o}}$	k_L_ (L/mg)	0.27
		Q_m_ (mg/g)	224.0
		R_L_	0.15
		MRE (%)	9.55
		SSE	671.97
		(R_adj_)^2^	0.9640
Freundlich	$Q_{e}=K_{F}C_{e}{}^{1/n}$	k_F_ (mg/g)/(mg/L)^1/n^	65.96
		1/n	0.37
		MRE (%)	2.95
		SSE	124.19
		(R_adj_)^2^	0.9933
Tempkin	$Q_{e}=B_{T}\ln(k_{T}C_{e})$ $B_{T}=\frac{RT}{b}$	k_T_ (L/mg)	3.17
		B_T_	46.22
		MRE (%)	6.23
		SSE	366.49
		(R_adj_)^2^	0.9804
D-R	$Q_{e}=Q_{m}\exp\left(-B\varepsilon^{2}\right)$ $\varepsilon =RT\ln\left(1+\frac{1}{C_{e}}\right)$ $E=\frac{1}{\sqrt{2B}}$	B (kJ^2^/mol^2^)	0.61
		Q_m_ (mg/g)	174.0
		E (kJ/mol)	0.91
		MRE (%)	11.82
		SSE	1900.35
		(R_adj_)^2^	0.8982

**Table 9:** Comparative maximum TCC adsorption capacity (Q_m_) using various materials.

No.	Adsorbents	Q_m_ (mg/g)	Ref.
1	MIL-53-derived mesoporous carbon (MPC)	224.0	This study (*)
2	Nanocrystalline cellulose	13.2	[57]
3	Alkali biochar	58.8	[69]
4	Pumice stone	20.0	[68]
5	g-C_3_N_4_ granules	70.0	[72]
6	Organic Acid-Coated Magnetic NPs	117.7	[55]
7	Ferric-activated SBA	87.9	[73]
8	Nanoscale zero-valent iron (nZVI)	105.5	[74]
9	Pumice modified nZVI	115.1	[74]
10	Carbons@carboxymethylcellulose	136.9	[75]
11	Reduced graphene oxide (RGO)	44.2	[70]
12	RGO-decorated Fe_2_O_3_ NPs	18.47	[70]
13	Biocomposites	11.2–18.3	[76]

## Supplementary Materials

1. Chemicals and instruments; 2. Mathematical formula; 3. Synthesis of MIL-53 (Fe) and MPC material; 4. Determination of pH_pzc_ (pH point of zero charges); 5. Error analysis; 6. Kinetic models; 7. Intraparticle diffusion; 8. Isotherm models; Figure S1. XRD diffraction MIL-53 (Fe) (a) and MPC (b); Figure S2. Raman spectra of MIL-53 (Fe) (a) and MPC (b); Figure S3. N_2_ adsorption/desorption measurement plots of MIL-53 (Fe) (a) and MPC (b); Figure S4. Actual versus predicted (a) and residuals versus runs (b) plots; Figure S5. Normal plot of residuals (a) and predicted values versus residuals (b) for the model of TCC removal; Figure S7. Cook’s distance (a) and Box-Cox plot for power transforms (b); Figure S8. Leverage versus run (a) and EFFITS versus run (b) plots; Table S1. Several properties of the TCC antibiotic (Source: Drugbank); Table S2. Matrix of observed and predicted values; Table S3. Confirmation test.
