# Supplementary material for: Combined Minimum-Run Resolution IV and Central Composite Design for Optimized Removal of the Tetracycline Drug Over Metal–Organic Framework-Templated Porous Carbon
Source: Molecules. 2019 May 16;24(10):1887. doi: 10.3390/molecules24101887 (PMC6571721; doi:10.3390/molecules24101887)
Supplement: Supplementary file 1 [file molecules-24-01887-s001.pdf]

Supplementary information

Combined minimum-run resolution IV and central composite design for optimized removal of tetracycline drug and over metal–organic framework-templated porous carbon

Thuan Van Tran,[1,2] Duyen Thi Cam Nguyen,[1,3] Hanh T. N. Le,[4] Long Giang Bach,[2,5] Dai-Viet N. Vo,[1,6] Kwon Taek Lim,[7] Linh Xuan Nong[1,2] and Trinh Duy Nguyen[1,2,*]

[1] Center of Excellence for Green Energy and Environmental Nanomaterials (CE@GrEEN), Nguyen Tat Thanh University, 300A Nguyen Tat Thanh, District 4, Ho Chi Minh City 755414, Vietnam

[2] NTT Hi-Tech Institute, Nguyen Tat Thanh University, 300A Nguyen Tat Thanh, District 4, Ho Chi Minh City 755414, Vietnam

[3] Department of Pharmacy, Nguyen Tat Thanh University, 298–300A Nguyen Tat Thanh, Ward 13, District 4, Ho Chi Minh City 700000, Vietnam

[4] Institute of Hygiene and Public Health, 159 Hung Phu, Ward 8, District 8, Ho Chi Minh City 700000, Vietnam.

[5]Center of Excellence for Functional Polymers and NanoEngineering, Nguyen Tat Thanh University, 300A Nguyen Tat Thanh, District 4, Ho Chi Minh City 755414, Vietnam

[6] Faculty of Chemical & Natural Resources Engineering, Universiti Malaysia Pahang, Lebuhraya Tun Razak, 26300 Gambang, Kuantan, Pahang, Malaysia

[7]Department of Display Engineering, Pukyong National University, Nam-Gu, Busan 608-737, Korea

Correspondence

* To whom correspondence should be addressed: T.D. Nguyen (Email: ndtrinh@ntt.edu.vn); Tel: (+84)-028-3941-1211 Fax: (+84)-028-39-404-759

1. Chemicals and instruments

All chemicals including tetracycline, 1,4–dicarboxylic acid (H_2BDC), iron chloride $FeCl_3.6H_2O$, and potassium chloride KCl were commercially purchased from Merck, and directly used without any purification method.

The D8 Advance Bruker powder diffractometer was used to record the X–ray powder diffraction (XRD) profiles using Cu–Kα beams as excitation sources. The S4800 instrument (Japan) was implemented to capture the scanning electron microscope (SEM) images with the magnification of 7000 using an accelerating voltage source (15 kV). The JEOL JEM 1400 instrument was used to study the transmission electron microscopy (TEM). The FT–IR spectra were recorded on the Nicolet 6700 spectrophotometer. The N_2 adsorption/desorption isotherm and pore size distribution data were recorded on the Micromeritics 2020 volumetric adsorption analyzer system. The mapping element

profiles were recorded on the JEOL JSM-7600F (USA). The UV–Vis spectrophotometer was used to determine the TCC concentration at 272 nm.

2. Mathematical formula

The kinetic and equilibrium adsorption capacities Q_t and Q_e (mg/g) were calculated by the following equations (Eq. S1, S2):

$$Q_t = \frac{C_o - C_t}{m}.V \quad (S1)$$

$$Q_e = \frac{C_o - C_e}{m}.V \quad (S2)$$

where, C_o, C_t, and C_e are initial, time and equilibrium concentrations (mg/L), respectively; m (g) and V (ml) are the amount of adsorbent and volume of solution, respectively.

3. Synthesis of MIL-53 (Fe) and MPC material

The production route of MIL-53 (Fe) was based on the previous work [1]. Firstly, 1.35 g of $FeCl_3.6H_2O$ and 0.83 g of H_2BDC were dissolved in 25 mL *N,N*-dimethylformamide (DMF). The mixture was then transferred into a Teflon-lined autoclave and heated up at 180 ^oC for 6 h. The solid was extracted, washed with C_2H_5OH for several times, dried at 110 ^oC, and used for the synthesis of MPC in the next procedure.

The MPC were produced by the direct pyrolysis of MIL-53 (Fe) as a self-sacrificial precursor. The procedure could be carried out as follows: 0.8 g of MIL-53 (Fe) was

carefully embarked on a heat-resistant vessel, and pyrolyzed at 800 ^oC for 4h under N_2 (100 cm^3/min). The as-received black samples were finally stored in a desiccator cabinet, ad denoted as MPC (MIL-53 (Fe)-derived mesoporous carbon).

4. Determination of pH_{pzc} (pH point of zero charges)

The pH_{pzc} determination was determined according to a previous report [2]. In a typical experiment, 5.0 mg of materials were poured into six flasks containing 25 mL of KCl 0.1 mol/L at the different pH values (pH_1 = 2, 4, 6, 8, 10, 12) adjusted using the HCl and NaOH solutions. The solutions were stirred for 10 minutes and maintained stable during 24 h. The solids were then separated from the mixture, and their final pH_2 were measured by a pH meter. The curve was plotted via pH_2 versus pH_1 and the pH_{pzc} was visualized at $pH_1 = pH_2$.

5. Error analysis

In the nonlinear kinetic and isotherm studies, error functions can be applied for the optimization process to compare the fitness between experimental and calculated data. Herein, three common error analysis functions were utilized to assess the nonlinear models including coefficient of determination (R^2), mean relative error (MRE), sum square error (SSE) in Eq. S3–S5. The kinetic and isotherm parameters were identified by minimizing the error functions over using the Origin ® 9.0 software (Massachusetts,

United States) [3]. Note that $Q_{i,cal}$ and $Q_{i,exp}$ are the theoretical and experimental values, respectively.

$$R^2 = \frac{\sum_{i=1}^{n}(Q_{i,\exp} - \overline{Q_{i,\exp}})^2 - \sum_{i=1}^{n}(Q_{i,\exp} - \overline{Q_{i,cal}})^2}{\sum_{i=1}^{n}(Q_{i,\exp} - \overline{Q_{i,\exp}})^2} \quad \text{(S3)}$$

$$MRE\,(\%) = \frac{100}{n}\sum_{i=1}^{n}\left|\frac{Q_{i,cal} - Q_{i,\exp}}{Q_{i,\exp}}\right| \quad \text{(S4)}$$

$$SSE = \sum_{i=1}^{n}\left(Q_{i,cal} - Q_{i,\exp}\right)^2 \quad \text{(S5)}$$

6. Kinetic models

In kinetic studies, we addressed several nonlinear models to evaluate the uptake and elucidate the factors affecting adsorption in heterogeneous phase. Therefore, adsorption of TCC onto MPC was simulated by kinetics models including pseudo first-order, pseudo second-order, Elovich and Bangham equations (Eq. S6–S10). By comparing with coefficient of correlation R^2, MRE, SSE values, their fitness was suggested to choose the most compatible model for the description on experimental and calculated data.

Initially, we applied the nonlinear kinetic model, namely “pseudo first-order model” to reveal the correlation between the rate of adsorption and the number of unabsorbed sites. The mathematical form of pseudo first-order model could be described as follows (Eq. S6) [4].

$$Q_t = Q_1.(1-\exp(-k_1 t)) \quad \text{(S6)}$$

Where, k_1 is the pseudo first-order rate constant (min^{-1}), Q_t (mg/g) is the adsorption capacity at the time t (min), and Q_1 (mg/g) is the adsorption capacity at the equilibrium time (min)

One the most common kinetic model proposed is that "pseudo second-order equation" (Eq. S4), which assumes that adsorption process is controlled by chemisorption mechanism. The mathematical form of pseudo second-order model could be described in Eq. S7:

$$Q_t = \frac{t}{\frac{1}{k_2 Q_2^2} + \frac{t}{Q_2}} \quad \text{(S7)}$$

$$H = k_2.Q_2^2 \quad \text{(S8)}$$

Where, k_2 (g/mg min) is pseudo first-order rate constant, Q_t (mg/g) is the adsorption capacity at the time t (min), Q_2 (mg/g) is the adsorption capacity at the equilibrium time (min), H (mg/g min) is initial adsorption rate, which is determined in Eq. S8 [5].

The nonlinear Elovich model (Eq. S9) is used to elucidate the chemisorption processes, which solid/gas adsorption is carried out through heterogeneous adsorbing surfaces [6,7]. The characteristic of this chemisorption is a reversible process between

adsorption and desorption, which are defined by the adsorption rate α (mg/g min) and the desorption rate β (g/mg).

$$Q_t = \frac{1}{\beta}\ln(1+\alpha\beta t) \text{ (S9)}$$

Meanwhile, the nonlinear Bangham equation (Eq. S10) is used to explain the adsorbate pore diffusion activities, where α_B and k_B are its constants [6].

$$Q_t = k_B.t^{\alpha_B} \text{ (S10)}$$

7. Intraparticle diffusion

The Weber–Morris intraparticle diffusion equation (Eq. S11), which assumes that the mechanism for TCC adsorption occurs the bulk, external mass transfer, or diffusion of TCC molecules through the micropores of MPC material and chemical reactions (adsorption/desorption) in heterogeneous phrases. Its mathematical form can be described as follows:

$$Q_t = k_i.t^{1/2} + C_i \text{ (S11)}$$

Where, Where, k_i is the intraparticle rate constant (mg/gmin$^{0.5}$) and C_i is the Weber–Morris constant (mg/g). The slope of plot of Q_t versus $t^{1/2}$ gives the value of the intraparticle rate constant [8].

8. Isotherm models

In this study, we carried out the experiments to assess the effect of initial TCC concentration (10–40 mg/L) at room temperature on the adsorption capacity, then experimental data were converted into the nonlinear forms, which were proposed by Langmuir, Freundlich, Temkin, and Dubinin-Radushkevich (D-R) models (Eq. S12 – Eq. S19). Similarly, their fitness was used to choose the most compatible model to explain the adsorption mechanism and the correlation between experimental and calculated data.

Firstly, Langmuir equation is used to describe the adsorption mechanism of TCC drug onto MPC via monolayer adsorption behavior [9]. The mathematical form of Langmuir equation could be described in Eq. S12.

$$Q_e = \frac{Q_m K_L C_e}{1 + K_L C_e} \quad \text{(S12)}$$

$$R_L = \frac{1}{1 + K_L C_o} \quad \text{(S13)}$$

Where, Q_e (mg/g) is the equilibrium adsorption capacity, C_e (mg/L) is the equilibrium TCC concentration, Q_m (mg/g) is the maximum adsorption capacity, K_L (L/mg) is the Langmuir constants.

Note that R_L, which is described in Eq. S13, is used to evaluate the favorability of a process according to the following description:

If	Comment
$R_L = 0$	An unfavorable process
$0 < R_L < 1$	A favorable process
$R_L = 1$	An irreversible process

Secondly, Freundlich isotherm is used to describe an adsorption process, which tends to occur the multilayer adsorption behavior of TCC onto MPC. This mechanism is suggested by adsorption of adsorbate on heterogonous phase surfaces without any uniform distribution of heat of energies [2]. The mathematical form of Freundlich equation could be described in Eq. S14.

$$Q_e = K_F C_e^{1/n} \quad \text{(S14)}$$

Where, K_F (mg/g)(L/mg)$^{1/n}$ is the Freundlich constant, and 1/n is the equation's coefficient. Also, they can be calculated via the intercept and slope of the Freundlich equation (Eq. S14).

Thirdly, we used the Temkin isotherm to study the influence of "indirect interactions" between TCC molecules and the surface of adsorbent on the adsorption process. The mathematical form of Temkin equation could be described in Eq. S15 [10].

$$Q_e = B_T \ln(k_T C_e) \quad \text{(S15)}$$

$$B_T = \frac{RT}{b} \quad \text{(S16)}$$

Where, B_T is the Temkin coefficient, Q_e (mg/g) is the equilibrium adsorption capacity, C_e (mg/L) is the equilibrium concentration, K_T (L/g) is the the constants of Temkin equation, and R is the gas constant (8.314 J/mol K) (Eq. S16).

Finally, to explain the state of chemical/physical adsorption, we used Dubinin and Radushkevich (D-R) isotherm (Eq. S17). The mathematical form of Temkin equation could be described in Eq. S17.

$$Q_e = Q_m \exp\left(-B\varepsilon^2\right) \text{ (S17)}$$

$$\varepsilon = RT \ln\left(1 + \frac{1}{C_e}\right) \text{ (S18)}$$

$$E = \frac{1}{\sqrt{2B}} \text{ (S19)}$$

Where, B (mol^2/kJ^{-2}) and Q_m (mg/g) are the D-R and activity coefficients, Polanyi potential Ɛ (kJ/mol) and energy of adsorption E (kJ/mol) can be calculated from Eq. S18 and Eq. S19.

References

[1] T. Van Tran, D.T.C. Nguyen, H.T.N. Le, T.T.K. Tu, N.D. Le, K.T. Lim, L.G. Bach, T.D. Nguyen, MIL-53 (Fe)-directed synthesis of hierarchically mesoporous carbon and its utilization for ciprofloxacin antibiotic remediation, J. Environ. Chem. Eng.

7 (2019) 102881. doi:https://doi.org/10.1016/j.jece.2019.102881.

[2] L.G. Bach, T. Van Tran, T.D. Nguyen, T. Van Pham, S.T. Do, Enhanced adsorption of methylene blue onto graphene oxide-doped XFe2O4 (X = Co, Mn, Ni) nanocomposites: kinetic, isothermal, thermodynamic and recyclability studies, Res. Chem. Intermed. 44 (2018) 1661–1687.

[3] J.S. Piccin, T.R.S. Cadaval, L.A.A. de Pinto, G.L. Dotto, Adsorption isotherms in liquid phase: experimental, modeling, and interpretations, in: Adsorpt. Process. Water Treat. Purif., Springer, 2017: pp. 19–51.

[4] S. Eris, S. Azizian, Analysis of adsorption kinetics at solid/solution interface using a hyperbolic tangent model, J. Mol. Liq. 231 (2017) 523–527.

[5] S. Eris, S. Azizian, Extension of classical adsorption rate equations using mass of adsorbent: A graphical analysis, Sep. Purif. Technol. 179 (2017) 304–308.

[6] K.Y. Foo, B.H. Hameed, Insights into the modeling of adsorption isotherm systems, Chem. Eng. J. 156 (2010) 2–10. doi:https://doi.org/10.1016/j.cej.2009.09.013.

[7] M.J.F. Jasni, M. Arulkumar, P. Sathishkumar, A.R. Mohd Yusoff, N.A. Buang, F.L. Gu, Electrospun nylon 6,6 membrane as a reusable nano-adsorbent for bisphenol A removal: Adsorption performance and mechanism, J. Colloid Interface Sci. 508 (2017) 591–602. doi:https://doi.org/10.1016/j.jcis.2017.08.075.

[8] B. Das, R.R. Devi, I.M. Umlong, K. Borah, S. Banerjee, A.K. Talukdar, Arsenic (III) adsorption on iron acetate coated activated alumina: thermodynamic, kinetics and equilibrium approach, J. Environ. Heal. Sci. Eng. 11 (2013) 42.

[9] T. Van Tran, Q.T.P. Bui, T.D. Nguyen, N.T.H. Le, L.G. Bach, A comparative study on the removal efficiency of metal ions (Cu2+, Ni2+, and Pb2+) using sugarcane bagasse-derived ZnCl2-activated carbon by the response surface methodology, Adsorpt. Sci. Technol. 35 (2017) 72–85.

[10] A. Zuorro, G. Maffei, R. Lavecchia, Kinetic modeling of azo dye adsorption on non-living cells of Nannochloropsis oceanica, J. Environ. Chem. Eng. 5 (2017) 4121–4127.

List of figures

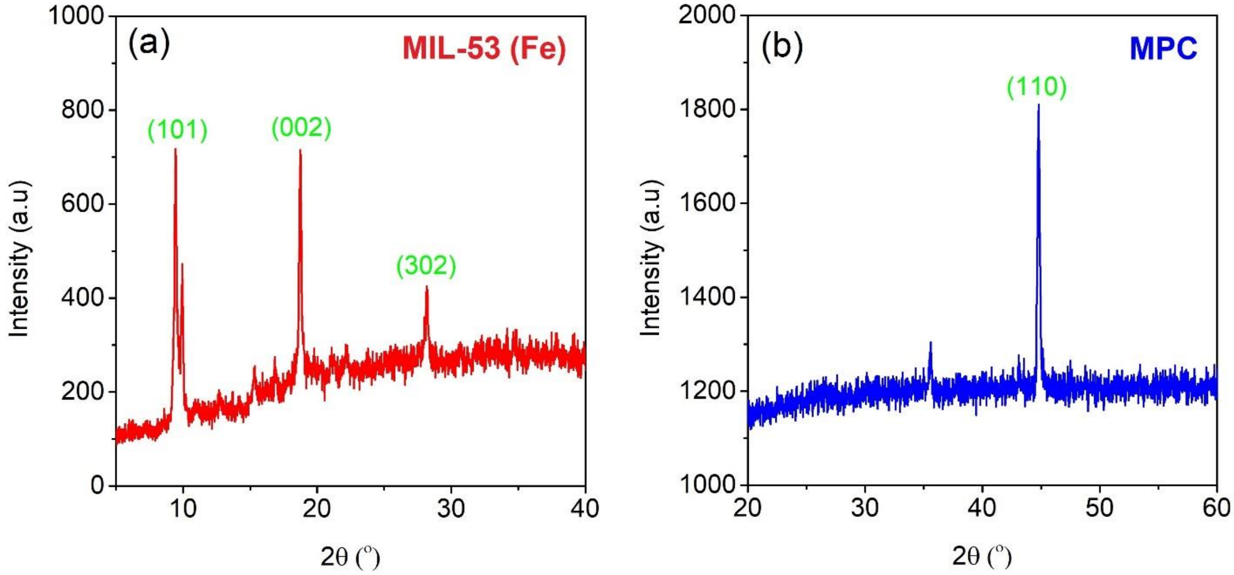

Figure S1. XRD diffraction MIL-53 (Fe) (a) and MPC (b)

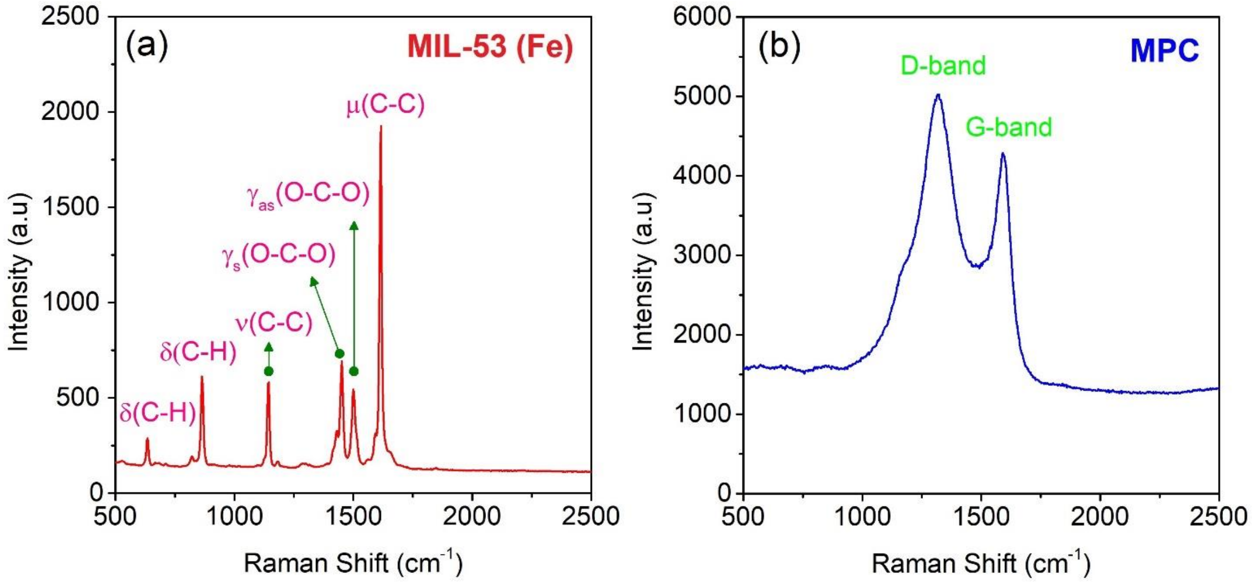

Figure S2. Raman spectra of MIL-53 (Fe) (a) and MPC (b)

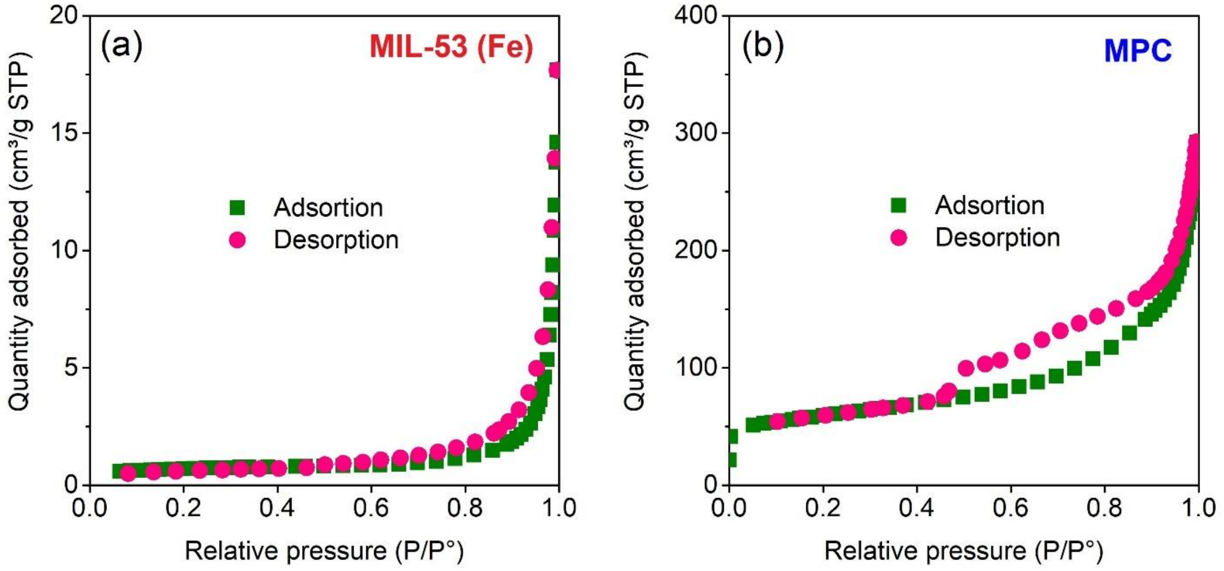

Figure S3. N_2 adsorption/desorption measurement plots of MIL-53 (Fe) (a) and MPC (b)

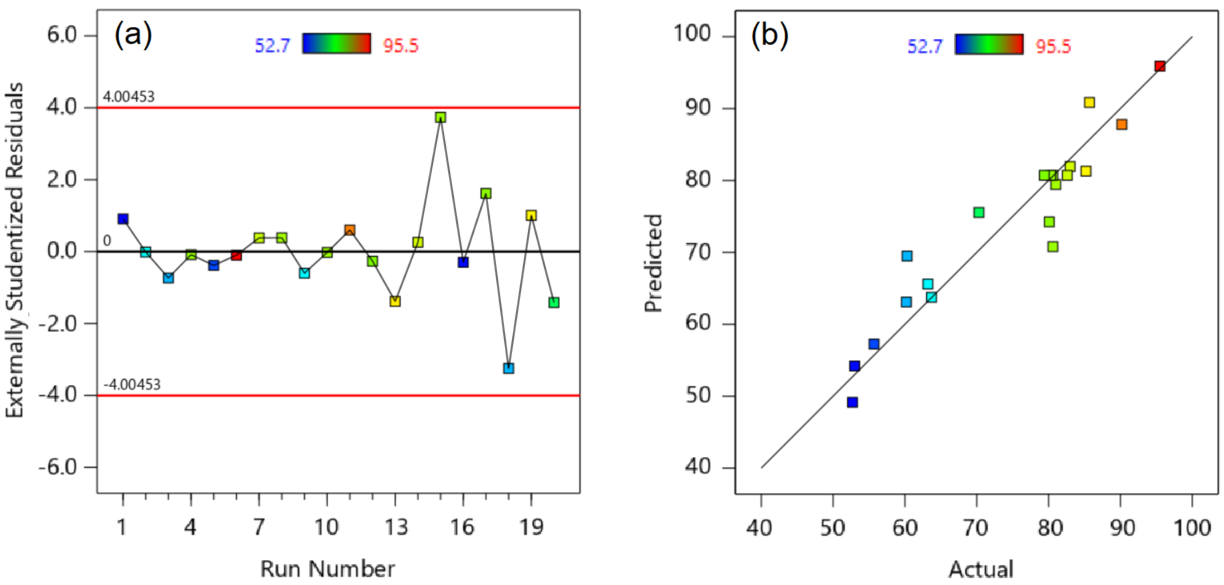

Figure S4. Actual versus predicted (a) and residuals versus runs (b) plots

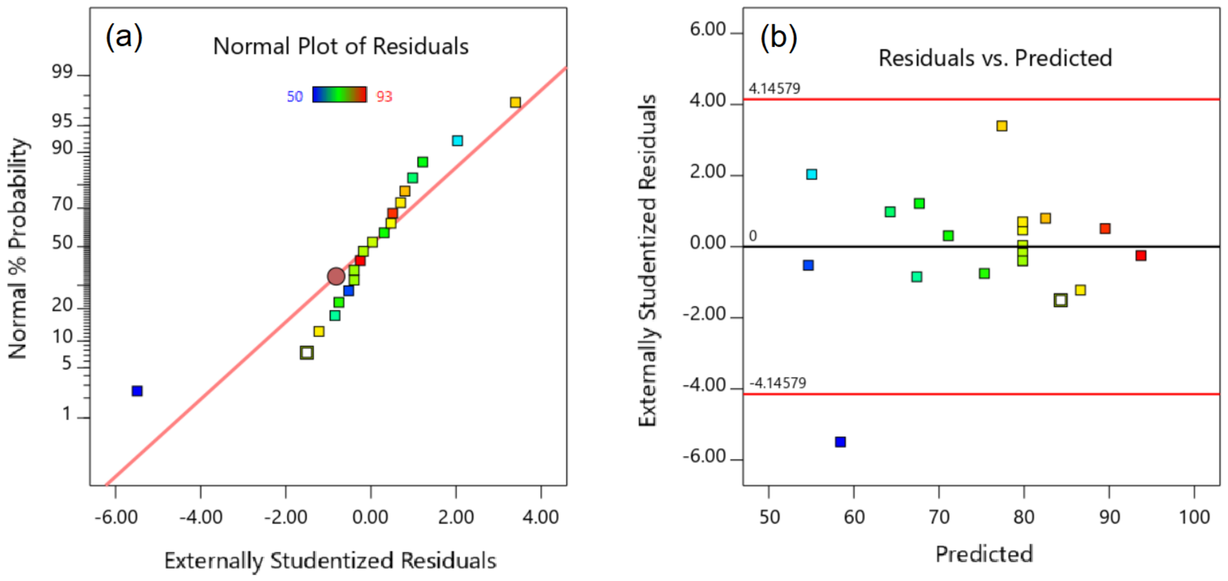

Figure S5. Normal plot of residuals (a) and predicted values versus residuals (b) for the model of TCC removal

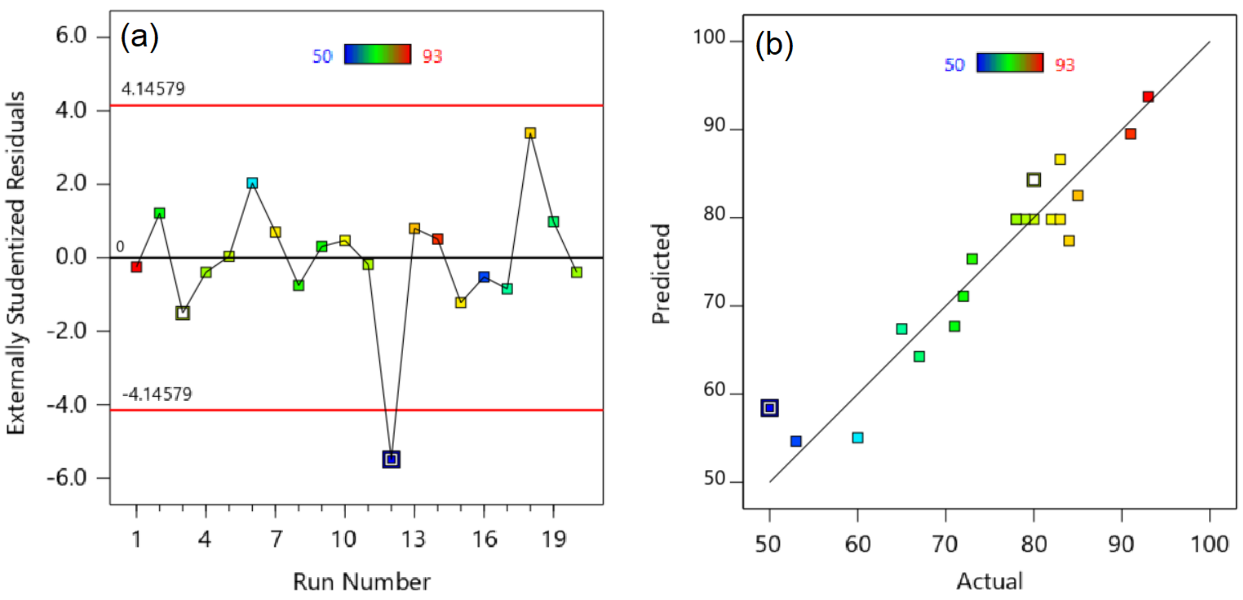

Figure S6. Residuals versus runs (b) and actual versus predicted (a) plots

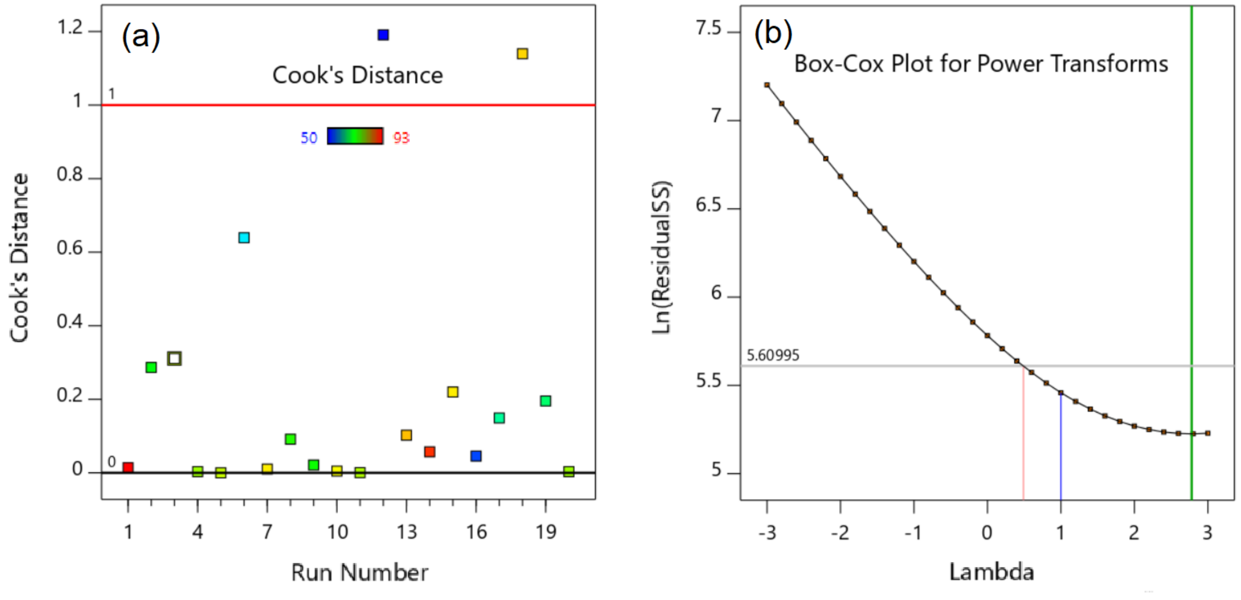

Figure S7. Cook's distance (a) and Box-Cox plot for power transforms (b)

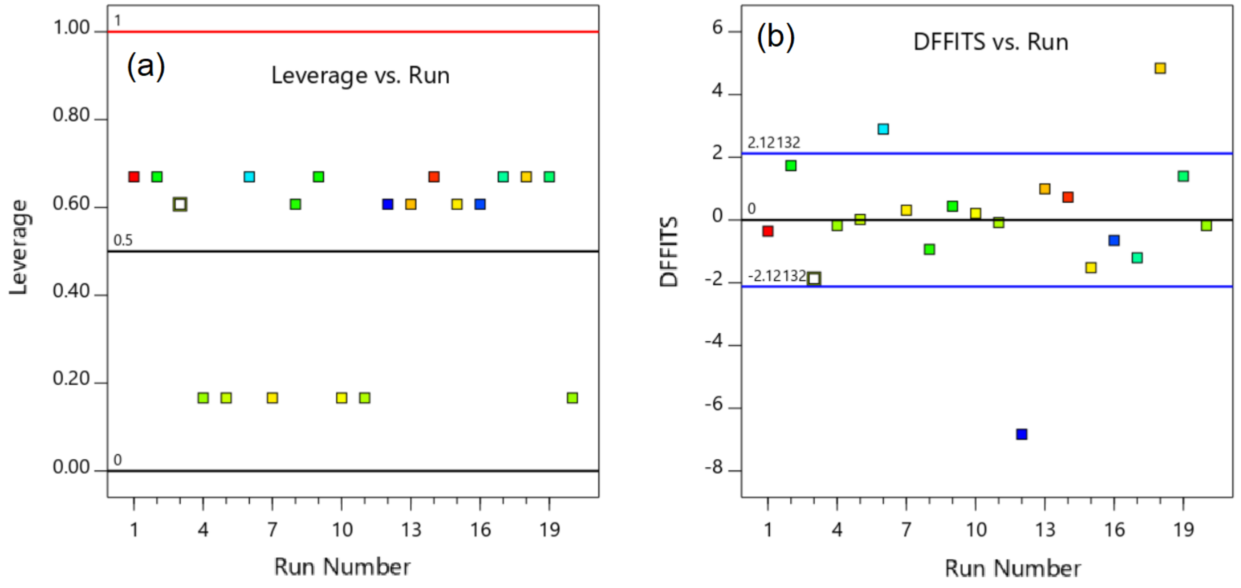

Figure S8. Leverage versus run (a) and EFFITS versus run (b) plots

List of Tables

Table S1. Several properties of TCC antibiotic (Source: Drugbank)

Molecular formula	Melting point (°C)	pK_a in water (25 °C)	Solubility in water (mg/L)	Wavelength (nm)	Number of H-bond
$C_{22}H_{24}N_2O_8$	172.5	$pKa_1 = 3.3$<br>$pKa_2 = 7.7$<br>$pKa_3 = 9.7$	231	272	17

Table 2. Matrix of observed and predicted values.

Run	Independent factors			TCC removal (%)	
	A (mg/L)	B (g/L)	C (-)	Actual	Predicted
1	5	0.05	4	84.0	77.4
2	15	0.05	4	71.0	67.7
3	5	0.15	4	93.0	93.7
4	15	0.15	4	91.0	89.5
5	5	0.05	8	67.0	64.3
6	15	0.05	8	60.0	55.1
7	5	0.15	8	72.0	71.1
8	15	0.15	8	65.0	67.4
9	1.6	0.1	6	83.0	86.6

10	18.4	0.1	6	73.0	75.3
11	10	0.016	6	50.0	58.4
12	10	0.184	6	85.0	82.5
13	10	0.1	2.6	80.0	84.3
14	10	0.1	9.4	53.0	54.7
15	10	0.1	6	80.0	79.8
16	10	0.1	6	78.0	79.8
17	10	0.1	6	79.0	79.8
18	10	0.1	6	82.0	79.8
19	10	0.1	6	78.0	79.8
20	10	0.1	6	83.0	79.8

Table S3. Confirmation test

No	Concentration (mg/L)	Dose (g/L)	pH (-)	Removal (%)			Desirability
				Predicted	Tested	Error	
1	1.9	0.15	3.9	96.0	99.7	+3.9	1.0000
2	5.9	0.16	4.0	93.3	98.0	+5.0	1.0000
3	5.1	0.13	3.8	93.4	93.4	0.0	1.0000
